# Astrocytes in Neurodegeneration: Spatial States, Crosstalk, and Emerging Therapies

**DOI:** 10.1007/s12035-026-06142-x

**Published:** 2026-09-14

**Authors:** Lisa Steger, Veit Rothhammer, Friederike Zunke

**Affiliations:** 1 Department of Molecular Neurology, University Hospital Erlangen, Friedrich-Alexander University Erlangen-Nürnberg (FAU), 91054 Erlangen, Germany; 2 Department of Neurology, University Hospital Erlangen, Friedrich-Alexander-University Erlangen-Nürnberg (FAU), 91054 Erlangen, Germany

**Keywords:** Astrocytes, Neurodegeneration, Spatial transcriptomics, Astrocyte heterogeneity

## Abstract

Astrocytes are increasingly recognized as active drivers of neurodegeneration rather than passive responders. Single-cell and spatial transcriptomic analyses reveal that astrocytes occupy heterogeneous, regionally patterned states that align closely with selective neuronal vulnerability. Across Alzheimer’s disease, Parkinson’s disease, ALS, Huntington’s disease, and rare primary astrocytopathies, astrocytes consistently converge on dysfunction across four mechanistic axes: breakdown of glutamate homeostasis, impaired ion and water buffering, lysosomal, and autophagic insufficiency, as well as maladaptive inflammatory-stress signaling. Spatial multi-omics demonstrates that these disruptions are not uniformly distributed but instead map to discrete niches, including plaque-adjacent astrocytes in Alzheimer’s disease, CD44-high fibrotic-like astrocytes in the substantia nigra in Parkinson’s disease, and EAAT2-low ventral horn astrocytes in ALS, consistent with patterns of selective neuronal vulnerability. Primary astrocytopathies including Alexander disease, vanishing white matter disease, and megalencephalic leukoencephalopathy illuminate the causal power of perturbing individual astrocytic modules, revealing how isolated disruptions in proteostasis, translation control, or ion-water coupling can initiate widespread neurodegeneration. By integrating neuropathological, imaging, and transcriptomic evidence across studies, we derive a consensus-based regional framework of astrocytic vulnerability across neurodegenerative diseases. Together, these findings define a unifying framework in which astrocytes transition from homeostatic regulators to pathological amplifiers, highlighting astrocyte states as tractable, region-specific therapeutic targets and illustrating how integration of spatial atlases with mechanistic insights might help develop a framework for targeted astrocyte therapies.

**Graphical Abstract:**

**Figure:**
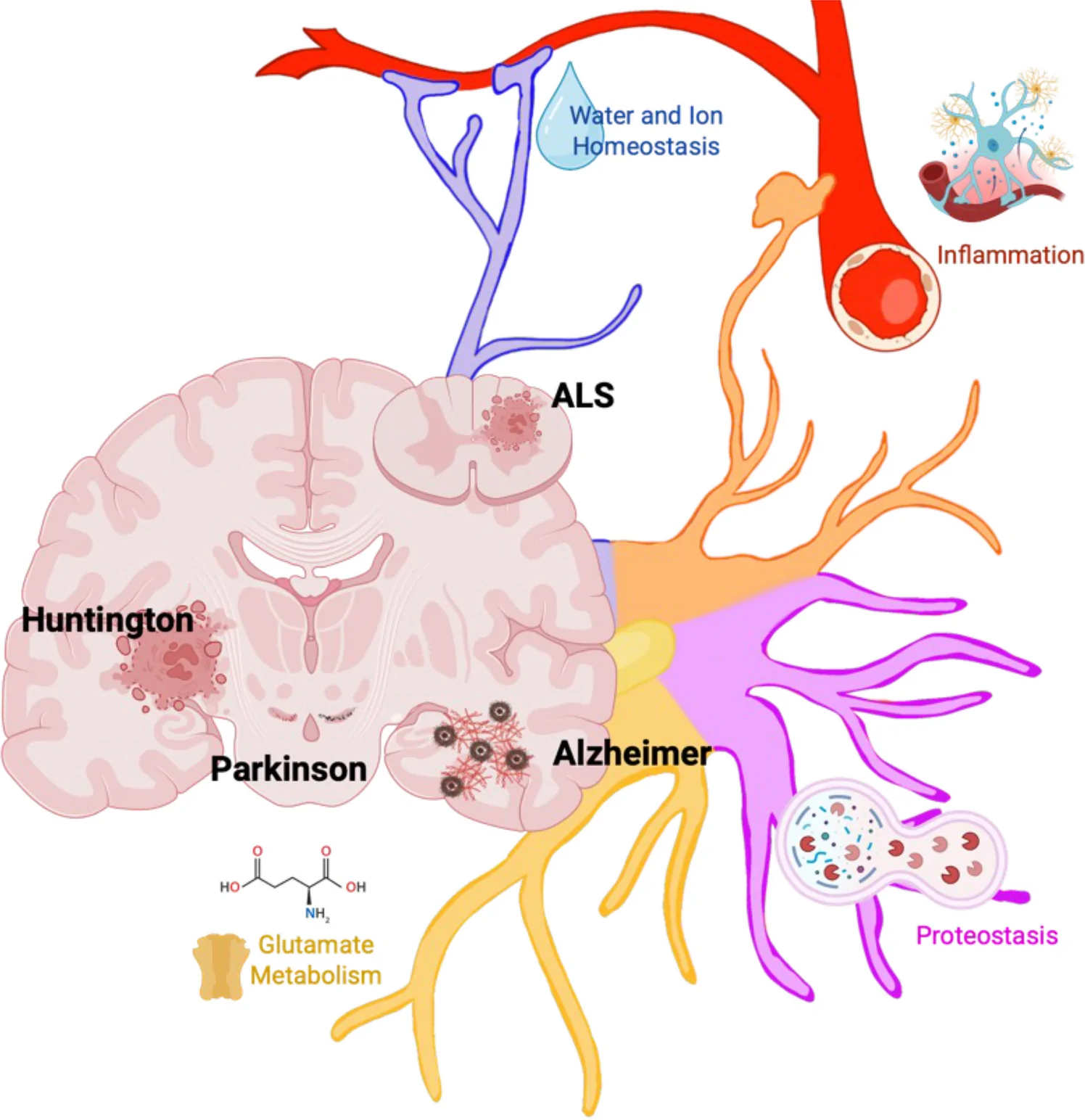

**Supplementary Information:**

The online version contains supplementary material available at https://doi.org/10.1007/s12035-026-06142-x.

## Background

**Fig. 1:**
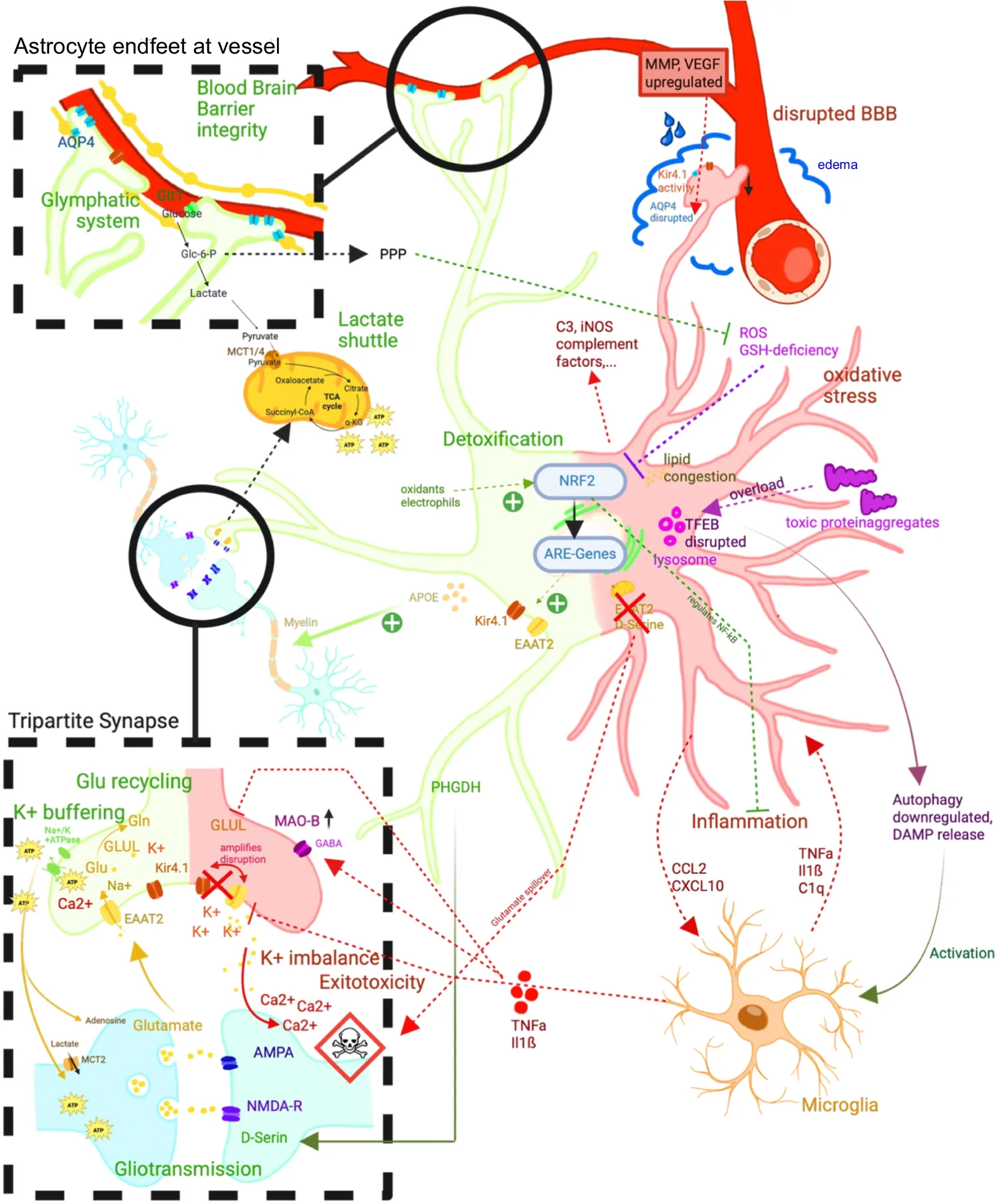
**Overview of astrocyte functions and dysfunctions.** Astrocytes maintain neuronal and vascular homeostasis by supporting neurotransmitter recycling, potassium and water buffering, lactate shuttling, detoxification, and blood-brain barrier integrity. These homeostatic functions are shown in green, with astrocytic endfeet at the vasculature and the tripartite synapse highlighted to illustrate their central roles in energy supply, glutamate clearance, and ionic balance. In disease, these mechanisms become maladaptive and actively drive pathology. Failures in Na⁺/K⁺-ATPase activity, Kir4.1/AQP4 polarity, and EAAT2 trafficking reduce glutamate uptake and K⁺/water flux, predisposing to excitotoxicity and edema. Impaired astrocyte-neuron lactate shuttling, GLUL activity, and ApoE-dependent trafficking further weaken metabolic support. Disruption of NRF2-ARE signaling lowers antioxidant capacity, while MAO-B-mediated H₂O₂ production enhances oxidative stress. Defects in TFEB-dependent lysosomal tone impair aggregate handling and promote release of DAMPs. In parallel, astrocytic secretion of complement, chemokines, and cytokines amplifies microglial activation and neuroinflammation

Although neurodegenerative diseases manifest as progressive and anatomically selective loss of neuronal populations, they share biological mechanisms that extend beyond disease-specific protein aggregates or degenerating circuits [1, 2]. Across disorders such as Alzheimer’s disease (AD), Parkinson’s disease (PD), amyotrophic lateral sclerosis (ALS), and Huntington’s disease (HD), a recurring constellation of cellular stressors - including impaired proteostasis, synaptic dysfunction, metabolic collapse, oxidative injury, and innate immune activation - creates a landscape in which non-neuronal cells become decisive regulators of neuronal fate [3, 4]. Among these, astrocytes have undergone conceptual reframing over time [5]: once characterized primarily by “reactive gliosis” and placed in a binary A1/A2 classification, astrocytes are now recognized as highly plastic integrators of synaptic, metabolic, vascular, and inflammatory signals [6–9]. Single-cell sequencing and spatial transcriptomics demonstrate that astrocytes adopt a wide range of transcriptional programs that vary across brain regions, disease stages, and microenvironmental niches [6, 10]. Crucially, these states are anatomically patterned: astrocytes encircling amyloid plaques exhibit lysosomal, complement, and metabolic signatures distinct from those in plaque-free cortex; nigral astrocytes exposed to α-synuclein (α-syn) show lysosomal stress and CD44-enriched phenotypes not observed in striatal territories; and ventral horn astrocytes in ALS exhibit profound EAAT2, Kir4.1, and Cx43 hemichannel dysregulation not found outside motor pools [10–14]. This spatial logic provides a coherent framework for understanding selective neuronal vulnerability [15]. Across neurodegenerative diseases, astrocytic dysfunction converges on four recurrent mechanistic axes: disturbances in glutamate homeostasis, impaired ion and water buffering, lysosomal and autophagic stress, and maladaptive inflammatory or oxidative signaling (Fig. 1). These axes arise repeatedly in AD, PD, ALS, and HD and are mirrored with striking clarity in primary astrocytopathies, like Alexander disease, vanishing white matter disease, and megalencephalic leukoencephalopathy, where perturbation of a single astrocytic module is sufficient to drive widespread degeneration [16, 17]. Spatial omics data reveal how these mechanistic modules assemble into niche-specific astrocyte states that integrate aggregate load, synaptic activity, metabolic stress, and microglial cues into locally restricted but pathologically consequential programs [10, 15]. This spatially anchored framework reshapes the interpretation of astrocyte reactivity and suggests targeted interventions that act not on a global “reactive” phenotype but on discrete astrocyte states in anatomically vulnerable territories.

In this review, we summarize astrocyte diversity in health, outline the convergent mechanistic axes of dysfunction that define astrocyte pathology across neurodegenerative diseases, integrate spatial atlas data that map these dysfunctions into specific anatomical niches, and discuss rare astrocytopathies that isolate the contribution of individual astrocytic pathways. We then translate these insights into a conceptual “atlas-to-action” framework that informs therapeutic design, biomarker development, and precision delivery strategies for astrocyte-targeted interventions [18–20].

## Main

### Astrocytes in Health

Astrocytes perform a multifaceted range of functions essential for neuronal and vascular homeostasis [21, 22]. During development, radial glial cells guide neuronal migration and later mature into diverse astrocyte subtypes that populate gray and white matter [23, 24]. Mature astrocytes extend endfeet enveloping cerebral vasculature, forming a perivascular interface crucial for blood-brain barrier integrity and neurovascular coupling [25–27]. Through Ca^2^⁺-dependent pathways, astrocytic endfeet regulate arteriolar tone by releasing vasoactive metabolites, matching local blood flow to synaptic activity [28–30]. In parallel, polarized AQP4 localization supports glymphatic clearance of interstitial solutes, including metabolic waste products and potentially neurotoxic proteins such as amyloid-β [31–33].

At synapses, astrocytes are integral to the tripartite synapse with pre- and postsynaptic elements, shaping synaptogenesis, synaptic pruning, and synaptic plasticity [34–37]. They control neurotransmitter levels via high-affinity glutamate transporters (EAAT2/GLT-1), which rapidly clear glutamate from synaptic spaces to prevent excitotoxicity while sustaining presynaptic transmitter pools [38, 39]. EAAT2 function is tightly coupled to astrocytic ionic gradients, particularly potassium handling via Kir4.1 channels, meaning metabolic integrity and Na⁺/K⁺-ATPase activity directly determine glutamate uptake capacity as illustrated in Fig. 1 [40, 41]. Astrocytic connexins, especially Cx43 and Cx30, further organize these processes at the network level by enabling gap junctional coupling between neighboring astrocytes, thereby supporting potassium spatial buffering, and metabolite redistribution across local astrocyte territories [40, 42, 43]. Astrocytes also release gliotransmitters after detection of neuronal activity such as ATP, D-serine, and glutamate in activity-dependent manners, modulating excitatory and inhibitory synaptic transmission [44–49].

Metabolically, astrocytes support neurons through the astrocyte-neuron lactate shuttle, converting glucose from the blood to lactate (see Fig. 1) in order to supply neuronal oxidative metabolism at the synapse [50–52]. Furthermore, astrocytes are the brain’s primary cholesterol source and distribute ApoE-containing lipoproteins that support synapse formation and membrane turnover [53, 54]. In addition, astrocytes maintain redox balance via NRF2-driven antioxidant pathways and help detoxify ammonia and other metabolic byproducts [55–60]. At baseline, astrocytes exert an anti-inflammatory influence, constraining microglial activation and fine-tuning complement signals involved in synaptic refinement. This broad portfolio of functions, depicted in Fig. 1, highlights the extent to which neuronal survival depends on astrocytic homeostasis. Importantly, many of these processes are energetically demanding and exquisitely sensitive to stress. Glutamate uptake, potassium buffering, water handling, and metabolite redistribution depend on continuous ATP-dependent maintenance of ionic gradients and on coordinated activity within astrocytic networks. This provides a rationale for why astrocytes become destabilized in neurodegenerative disease.

### Mechanisms of Astrocyte Dysfunction in Neurodegeneration

Across neurodegenerative diseases, astrocytes fail in ways that reflect both their centrality to homeostasis and the vulnerabilities inherent to their physiology. First, the breakdown of glutamate transport is among the most consistent disturbances. EAAT2 expression and localization decline under inflammatory, oxidative, or metabolic stress, leading to extracellular glutamate accumulation and spillover onto extrasynaptic glutamate receptors, which trigger Ca^2^⁺ overload and excitotoxic signaling [61–64]. These glutamatergic deficits arise across AD, ALS, HD, and, to a lesser degree, PD, and represent a core mechanism linking astrocytic failure to neuronal death [3, 65].

Second, ionic and osmotic homeostasis deteriorates through disruption of Kir4.1 and AQP4 channels. Loss of Kir4.1 reduces K⁺ buffering capacity, elevating extracellular potassium and driving neuronal depolarization [32, 66–68]. AQP4 depolarization away from perivascular endfeet impairs glymphatic clearance, worsens edema, and destabilizes blood-brain barrier interactions [31, 69]. Both channels are co-regulated through the dystrophin-associated complex, and failure of either disrupts the synergy between water and ion homeostasis [70]. Also, metabolic coupling failures further destabilize astrocyte-neuron interaction as well as blood-brain barrier integrity by disrupting the ATP and ion gradients required for EAAT2 and Kir4.1 [53, 71]. Consistent with this broader network-level dependence, loss of astrocytic connexins Cx43 and Cx30 disrupts gap junctional coupling and impairs neuronal homeostasis, synaptic plasticity, and cognition [72].

Third, proteostasis and organelle homeostasis represent another major axis of failure [73]. Lysosomal biogenesis and autophagic flux diminish with chronic stress, often due to impaired TFEB signaling or direct genetic lesions such as Glucocerebrosidase (GBA1) mutations [74–77]. Lysosomal pH alterations and defects in cathepsins or glucocerebrosidase compromise lysosomal degradation of aggregated proteins, which accumulate and further impair trafficking and signaling [78–81]. Mitochondrial dysfunction adds to the burden by generating reactive oxygen species, impairing ATP production, and releasing mitochondrial DNA that activates cGAS-STING-driven inflammation [82]. Fourth, maladaptive inflammatory and oxidative-stress signaling represents a final convergent axis of astrocyte dysfunction. In some contexts, increased astrocytic MAO-B activity generates tonic GABA, while protective NRF2 programs are blunted [6, 57, 58, 83, 84], pointing out the interactions between metabolic, proteostatic, and inflammatory stress pathways. In parallel, NF-κB, JAK/STAT3, MAPK, and innate immune sensors such as TLRs and NLRP3 shift astrocytes toward reactive phenotypes that release cytokines, chemokines, and complement components [6, 7, 85, 86]. These signals act on microglia, endothelial cells, and neurons, creating self-reinforcing loops that maintain local inflammation even after initial triggers dissipate.

Furthermore, crosstalk across the neurovascular unit and with microglia further shapes the trajectory of dysfunction. BBB breakdown introduces direct contact with serum proteins such as albumin, which activate astrocytic TGF-β signaling [9, 87–89]. ATP leakage through hemichannels recruits microglia that drive astrocytes into complement-rich reactive states mediated by IL-1α, TNF, and C1q [90–92]. Neuronal hyperexcitability and metabolic stress feed back onto astrocytes, exacerbating demands on glutamate and potassium homeostasis. These distributed but coordinated failures illustrate why astrocyte pathology can be both a driver and amplifier of neurodegeneration.

### Astrocyte Dysfunction Across Neurodegenerative Diseases

Although AD, PD, ALS, and HD differ in genetics, age at onset, and clinical course, astrocytic contributions converge on a shared set of pathological programs. Across diseases, astrocytes repeatedly show impairment on the aforementioned four axes. Importantly, each disorder expresses a distinct combination and relative contribution of these pathways within defined anatomical niches, consistent with atlas-level and consensus frameworks describing modular, co-regulated astrocyte states.

**Fig. 2:**
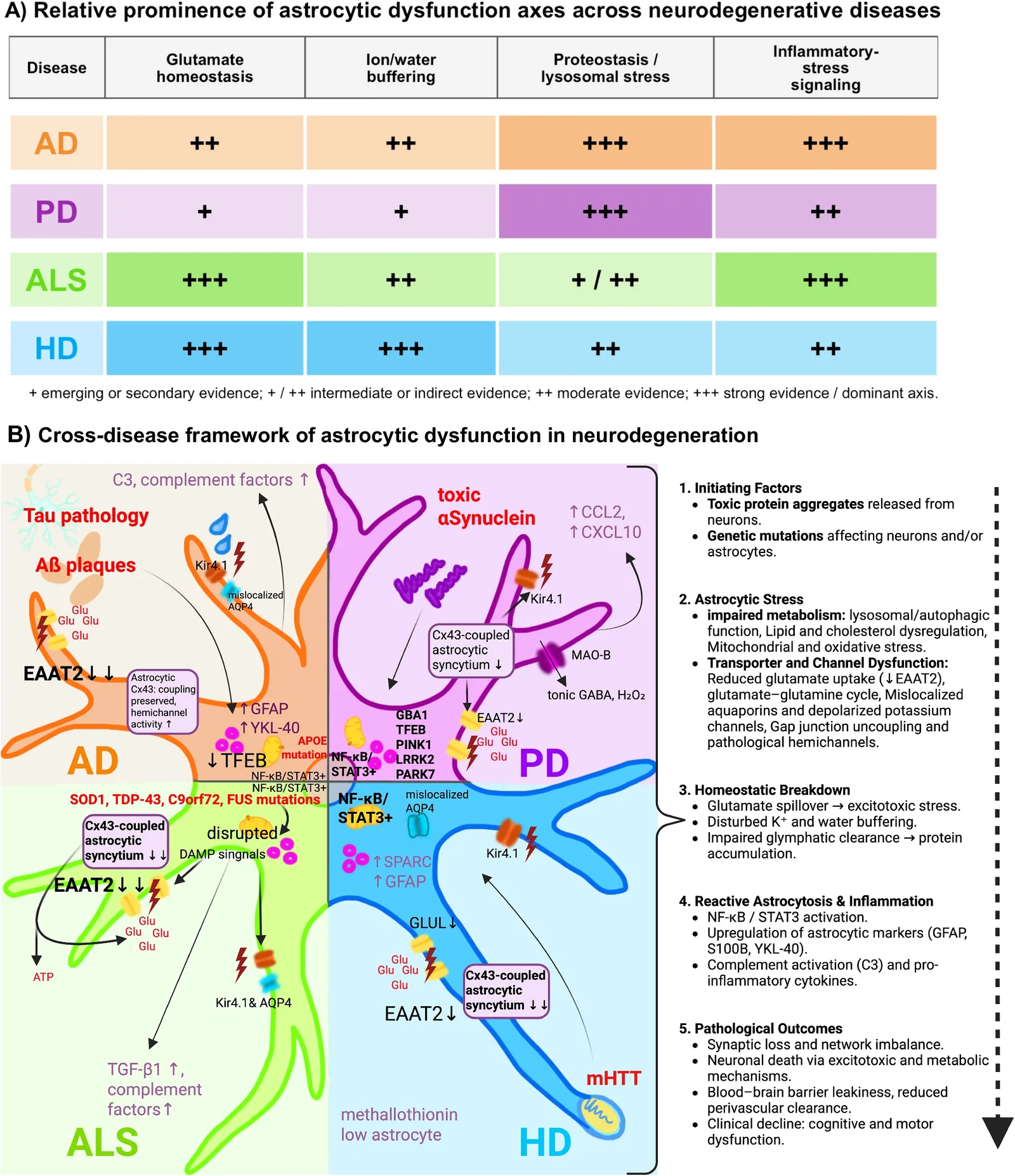
**Astrocytes at the crossroads: shared and disease-specific dysfunction in neurodegeneration.** **A)** Relative prominence of recurrent astrocytic dysfunction axes across major neurodegenerative diseases: heatmap summarizing the qualitative strength of evidence for four recurrent astrocytic dysfunction axes in Alzheimer’s disease (AD), Parkinson’s disease (PD), amyotrophic lateral sclerosis (ALS), and Huntington’s disease (HD). The grading reflects the relative prominence of each axis within the respective disease context based on consistency, disease relevance, and mechanistic support from the cited literature. “+” indicates emerging or secondary evidence, “ + + ” indicates moderate or recurrent evidence, and “ + + + ” indicates strong evidence and/or a disease-dominant axis. The categories are intended as qualitative and should not be interpreted as quantitative effect sizes or the result of a formal meta-analysis (see Supplementary Methods and Table 1). **B)** Cross-disease framework of astrocytic dysfunction in neurodegeneration: AD, PD, ALS, and HD. Schematic overview of recurrent astrocytic failure modes across Alzheimer’s disease (AD, orange), Parkinson’s disease (PD, purple), amyotrophic lateral sclerosis (ALS, green), and Huntington’s disease (HD, blue). Each quadrant highlights disease-specific triggers (toxic protein aggregates, genetic mutations) and converging astrocytic pathomechanisms, including impaired glutamate transport (↓EAAT2), mislocalized ion/water channels (Kir4.1, AQP4), lysosomal and autophagic stress (↓TFEB, GBA1 mutations), and inflammatory signaling (NF-κB, STAT3, complement, cytokines). These dysfunctions result in homeostatic breakdown, glutamate spillover, disturbed K⁺/water buffering, impaired glymphatic clearance, culminating in neuronal loss, blood-brain barrier disruption, and clinical decline

Clinical trajectories mirror these astrocytic axes: slowly accumulating metabolic and clearance deficits dominate in AD with plaque-associated inflammation present [10, 18], whereas rapidly evolving excitotoxic and inflammatory astrocyte states characterize ALS reflecting highly disrupted glutamate transport [61, 93], with PD and HD occupying intermediate, regionally constrained patterns [4, 94, 95] (Fig. 2A). Rare monogenic astrocytopathies serve as natural experiments that isolate single axes: proteostasis (Alexander disease), translational stress (vanishing white matter disease), ion-water homeostasis (megalencephalic leukoencephalopathy), or lysosomal failure (neuronal ceroid lipofuscinoses) and thereby validating the causal sufficiency of astrocyte dysfunction to drive neurodegeneration [96–101].

Together, these shared modules provide a mechanistic cross-disease framework for astrocytic states, identify convergent biomarkers and intervention points (e.g., EAAT2 trafficking, Kir4.1/AQP4 polarity, lysosomal homeostasis/function, NRF2 competence), and explain how vascular and microglial crosstalk stabilizes or amplifies chronic pathology. Building on this conceptual structure (Fig. 2), the following disease-specific pathways examine how these astrocytic modules are instantiated in Alzheimer’s disease, Parkinson’s disease, ALS, Huntington’s disease, and primary astrocytopathies.

### Astrocyte Dysfunction in Alzheimer’s Disease

AD is a progressive neurodegenerative disorder clinically characterized by episodic memory impairment, followed by deficits in executive function, language, and visuospatial abilities, ultimately leading to global cognitive decline and loss of independence [102]. Astrocytic dysfunction has been long recognized as a central contributor to AD pathogenesis, with astrocytes undergoing profound shifts tightly linked to amyloid deposition and tau pathology [102–104]. Plaque-associated astrocytes show repression of EAAT2, leading to glutamate spillover and aberrant Ca^2^⁺ signaling in nearby neurons [10, 105], depicted in Fig. 2. Their lysosomal compartments become overloaded with amyloid fragments, reflecting impaired TFEB-mediated lysosomal biogenesis [18, 106]. Plaque-associated astrocytes exert strong inflammatory effects: they express complement components such as C3 that mark synapses for elimination and secrete cytokines and chemokines that amplify microglial activation [7, 90]. At the vascular interface, AQP4 depolarization disrupts glymphatic clearance, promoting further amyloid accumulation [31, 107]. Together, these spatially confined astrocytic states contribute to circuit instability, metabolic stress, and synaptic loss [2, 108]. These changes are mirrored in fluid and imaging biomarkers such as GFAP, YKL-40, and MAO-B PET and p-tau assays, underscoring the translational relevance of astrocyte biology [109–111].

### Astrocyte Dysfunction in Parkinson’s Disease

PD is clinically defined by bradykinesia, rigidity, and rest tremor, later joined by gait and postural instability as well as prominent non-motor features [4, 112, 113]. Pathologically, Lewy bodies containing aggregated α-syn are a hallmark [114], and the progressive loss of nigrostriatal dopamine neurons follows a stereotyped caudo-rostral progression captured by Braak staging [115]. Astrocytic pathology centers on lysosomal dysfunction, inflammatory activation, with secondary disturbances in metabolic and neurotransmitter homeostasis [116]. Astrocytes internalize neuronal α-syn but degrade it inefficiently, particularly in the context of GBA1, ATP13A2, or DJ-1 mutations affecting lysosomal and cellular stress pathways [76, 77, 117–120]. Accumulated α-syn impairs lysosomal integrity and activates NF-κB pathways, generating chemokine-rich inflammatory states [7, 116]. Elevated MAO-B activity in disease-associated astrocytes increases tonic GABA release and hydrogen peroxide, suppressing neuronal excitability and contributing to synaptic dysfunction [84], illustrated in Fig. 2. Protective mechanisms such as NRF2-dependent glutathione metabolism are blunted in PD astrocytes, reducing antioxidant capacity [57, 58]. EAAT2 downregulation in astrocytes and impaired EAAT2 membrane trafficking further elevate extracellular glutamate, adding to dopaminergic stress [121, 122]. Altered AQP4 polarization, disrupted Kir4.1 activity, and impaired glymphatic function altogether implicated in α-syn propagation and neurodegeneration in PD [123, 124]. These disturbances concentrate in substantia nigra territories where dopaminergic neurons are most vulnerable, and spatial transcriptomic studies identify CD44-high astrocyte niches corresponding to these high-stress environments [13, 125].

### Astrocyte Dysfunction in ALS

ALS is a relentlessly progressive disorder characterized clinically by weakness with spinal or bulbar onset, upper motor neuron signs including spasticity and hyperreflexia, and eventual respiratory failure. Beyond motor involvement, patients frequently experience non-motor features such as cramps, weight loss, and cognitive or behavioral changes along the ALS-frontotemporal dementia (FTD) spectrum [126]. It features some of the most robust evidence for astrocyte-driven degeneration [127, 128]. Severe EAAT2 loss in ventral horn astrocytes is among the most robustly characterized astrocytic abnormalities and directly contributes to excitotoxic death of motor neurons [61, 129]. Kir4.1 dysfunction, gap junction uncoupling, and high Cx43 hemichannel activity lead to elevated extracellular K⁺ and aberrant ATP and glutamate release, creating para-excitatory microenvironments around motor neurons [93, 130]. Astrocytes accumulate misfolded SOD1, TDP-43, or C9orf72-derived dipeptide repeats, which disrupt lysosomal trafficking and autophagic flux [131, 132]. Inflammatory signaling through NF-κB, STAT3, and interferon pathways is upregulated, and astrocytes secrete complement, cytokines, and TGF-β1 that accelerate neuronal degeneration [5, 133]. Spatial atlases reveal that these pathological states are most intense near degenerating motor neurons and corticospinal pathways rather than diffusely distributed across spinal cord tissue [131] (Fig. 2).

### Astrocyte Dysfunction in Huntington’s Disease

HD is clinically defined by chorea and dystonia, psychiatric and behavioral disturbances, and a subcortical cognitive profile with progressive executive and memory decline [134]. Cognitive and affective symptoms often manifest years before motor onset, and standardized assessments such as the Unified Huntington’s Disease Rating Scale (UHDRS) enable longitudinal tracking [135]. In HD, astrocytes in the dorsal striatum and caudate nucleus experience profound deficits in glutamate and potassium homeostasis [136, 137]. Mutant huntingtin reduces EAAT2 expression and surface localization, impairs glutamine synthetase, and disrupts Na⁺/K⁺-ATPase coupling. Kir4.1 suppression leads to potassium accumulation and increased membrane excitability [68, 138, 139] (Fig. 2). Lysosomal and autophagic impairments hinder the clearance of huntingtin aggregates within astrocytes, amplifying intracellular stress [140]. Inflammatory activation through STAT3 and NF-κB pathways emerges in striatal regions with the highest neuronal vulnerability [141]. Strikingly, protective metallothionein-high astrocytes, enriched in cortical regions, are underrepresented in the striatum, suggesting that astrocyte heterogeneity contributes directly to selective neuronal loss [11, 137, 142].

### Primary Astrocytopathies

Rare monogenic astrocytopathies reveal how single-gene disruptions in astrocytes can drive widespread neurodegeneration, confirming the role of shared pathology mechanisms. Alexander disease (AxD), caused by dominant mutations in GFAP, leads to accumulation of Rosenthal fibers, severe proteostasis stress, and white-matter degeneration [17, 96]. Vanishing white matter disease (VWM), resulting from eIF2B mutations, exposes the vulnerability of astrocytes to disruptions in the integrated stress response; white matter deteriorates rapidly following metabolic insults such as fever or trauma [143]. Megalencephalic leukoencephalopathy (MLC), involving mutations in MLC1 or HEPACAM, disrupts ion and water regulation, leading to myelin vacuolation and macrocephaly [99]. Neuronal ceroid lipofuscinoses (Batten disease) represent monogenic lysosomal disorders in which CLN mutations disrupt lysosomal-autophagy pathways in neurons and astrocytes, causing storage pathology, neuroinflammation, and progressive neurodegeneration across diverse clinical phenotypes [100, 144–146]. These astrocytopathies demonstrate that disturbances in cytoskeletal stability, protein translation, or ion-water homeostasis within astrocytes are sufficient to initiate global neurodegenerative cascades.

### Spatial Omics Reveal Niche-Specific Astrocyte States

**Fig. 3:**
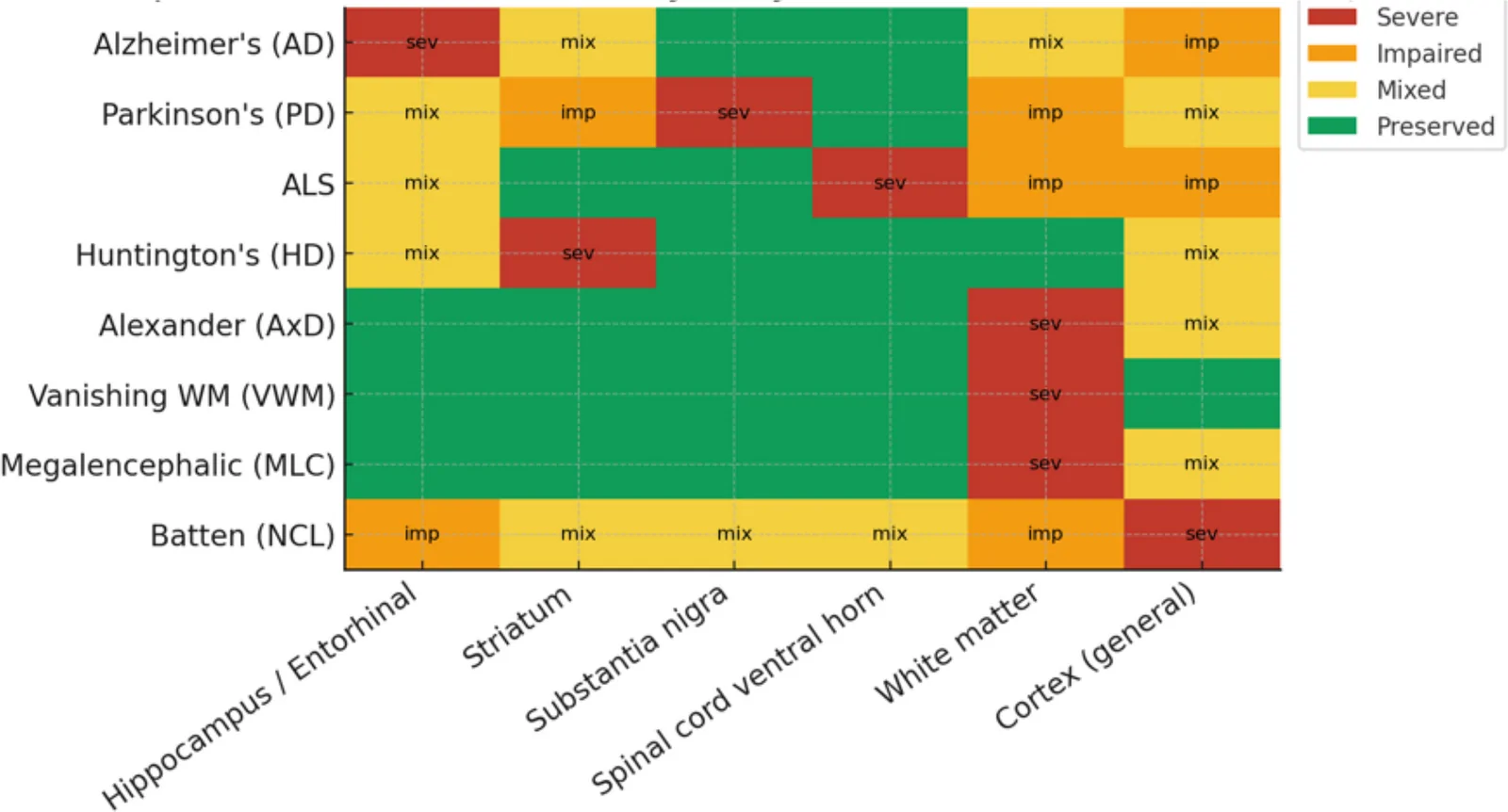
**Regional specificity of astrocytic dysfunction across neurodegenerative and primary astrocytic disorders.** Heatmap summarizing the severity of astrocytic impairment across major brain regions (sev, severe; imp, impaired; mix, mixed; green, preserved). Regional classifications were derived from peer-reviewed neuropathological, imaging, and transcriptomic studies referenced in Supplementary Table 2. Brain regions were categorized as “severe,” “impaired,” “mixed,” or “preserved” based on the consistency and strength of evidence for astrocytic dysfunction or regional pathology reported across studies. “Severe” indicates robust and reproducible evidence of astrocytic dysfunction or neuronal vulnerability in the respective region; “impaired” reflects moderate but consistent alterations; “mixed” indicates heterogeneous or context-dependent findings, and “preserved” reflects absence of consistent regional involvement in the cited literature. Classifications were qualitative and consensus-based among the authors and were not derived from quantitative meta-analysis. The severity categories thus indicate relative regional involvement rather than a binary distinction between functional and dysfunctional astrocytes. In multifactorial disorders, astrocytic dysfunction aligns with neuronal vulnerability: hippocampus and cortex in Alzheimer’s disease (AD), substantia nigra in Parkinson’s disease (PD), ventral horn in amyotrophic lateral sclerosis (ALS), and striatum in Huntington’s disease (HD). In primary astrocytopathies, regional specificity reflects the genetic lesion: Alexander disease (AxD), vanishing white matter (VWM), and megalencephalic leukoencephalopathy (MLC) primarily affect white matter, while Batten disease (NCL) shows a mixed cortical–white matter pattern. This highlights how spatial context dictates whether astrocytic dysfunction amplifies neuronal degeneration or drives pathology directly. Regional classifications were derived from qualitative analysis of neuropathological, imaging, and transcriptomic studies describing the dominant astrocytic dysfunction patterns and their regional distribution, including inflammatory plaque-associated states in AD, lysosomal/CD44-high states in PD, EAAT2/Kir4.1/Cx43-associated excitotoxic states in ALS, and glutamate-dominated striatal states in HD (see Supplementary Methods and Table 2)

The emergence of spatial transcriptomics and proteomics has transformed the understanding of astrocyte biology by revealing that astrocytic states are spatially patterned and intimately tied to microenvironmental context. Rather than reacting uniformly across affected brain regions, astrocytes form sharply demarcated niches of dysfunction. By “niche-specific astrocyte states,” we aim to describe these locally patterned, disease- and region-dependent combinations of shared astrocytic dysfunction modules, rather than discrete uniform astrocyte subtypes. Figure 3 summarizes these region-specific astrocytic states based on qualitative synthesis of neuropathological, imaging, and spatial omics studies (see Supplementary Information and Methods). In AD, plaque-adjacent astrocytes co-localize with microglial modules, a pattern recently corroborated at protein level in human AD tissue [147], and adopt complement/chemokine signaling, lysosomal TFEB activation, lipid and amino acid metabolic rewiring, and elevated calcium dynamics that reported to decrease with distance from plaques [10, 31, 105, 107, 148, 149]. Recent spatial transcriptomic analyses of amyloid plaque niches further refined this concept by showing that increasing microglial density is associated with a shift toward neurotoxic astrocyte states, emphasizing the role of intercellular crosstalk in disease associated niches [150]. The spatial progression of AD is well characterized by the Braak staging scheme, initially affecting the entorhinal cortex, where it is most severe, and subsequently spreading to neocortical regions and eventually to white matter and striatal structures [104, 151–153]. In PD, substantia nigra astrocytes exposed to dense α-syn loads exhibit lysosomal-stress transcriptional signatures and CD44-high fibrotic-like phenotypes, while neighboring striatal astrocytes show different stress modules shaped by denervation rather than α-synuclein accumulation [13, 81, 125]. White matter alterations are also frequently reported, while cortical and hippocampal involvement rather varies across patients [13, 81, 115, 154–157]. In ALS, ventral horn astrocytes demonstrate pronounced EAAT2 and Kir4.1 loss, high Cx43 hemichannel activity, and inflammatory signaling linked to degenerated motor neurons [93, 130]. ALS pathology can extend beyond the ventral horn to include widespread white matter degeneration, cortical phenotypes, and hippocampal involvement within the ALS-FTD spectrum [158–161]. In HD, dorsal striatal astrocytes display EAAT2 suppression, Kir4.1 deficits with fibrous (CD44-high) states while metallothionein-high islands correlate with relative neuronal preservation, whereas cingulate and accumbens astrocytes show comparatively preserved homeostatic modules [68, 136, 137, 139]. In addition, distinct motor phenotypes are associated with cortical thinning [162, 163], as well as hippocampal involvement is discussed [164]. These findings emphasize that astrocyte dysfunction cannot be meaningfully interpreted without spatial context. These spatially localized dysfunctions reveal that astrocyte states emerge from local microenvironmental demands: exposure to protein aggregates, proximity to degenerating neurons, vascular stress, and interactions with activated microglia. This spatial logic predicts where therapeutic interventions might need to be targeted to achieve maximal benefit.

### Astrocyte-Targeted Therapeutics: Opportunities and Barriers

Astrocyte-directed therapies are advancing into clinical and preclinical development: antisense oligonucleotides directed against GFAP (zilganersen) represent the first clinical program explicitly designed to modulate an astrocytic gene (NCT04849741) [165]. Preclinical gene-, vector-, and pharmacological approaches have restored selected astrocytic pathways aligned with the mechanistic axes in animal models, namely astrocytic Kir4.1 delivery in Huntington’s disease mice improved ion homeostasis, prolonged survival, and attenuated motor phenotypes [68]. Nevertheless, translation to humans remains limited and depends on achieving reliable astrocyte tropism and safe dosing [166]. Astrocyte-enriched targets such as MAO-B, which becomes upregulated in disease-associated astrocytes, are being pursued in reversible MAO-B inhibitors like KDS2010 [167]. More broadly, therapies that modulate inflammatory signaling, antioxidant pathways, or metabolic coupling are increasingly recognized as potential interventions [58, 168–171]. Two bottlenecks hamper translation: first, the challenge of delivering therapeutics across the BBB with sufficient astrocyte specificity, and second, the need for biomarkers that accurately reflect astrocytic engagement, including plasma GFAP, YKL-40, and imaging measures such as TSPO or MAO-B PET [20, 172–176]. The future of astrocyte-targeted therapy will likely require integrating spatial signatures as pharmacodynamic readouts.

### How Spatial Context Guides Clinical Translation

Spatial omics has shifted astrocyte biology from a descriptive to a decisively operational science, by revealing not only *which* astrocyte states exist but *where* they arise, *which* signals shape them, and *which* neuronal populations they endanger [8, 10, 15]. The challenge now is to convert this atlas-level knowledge into therapies delivered to the right place, at the right time, and in the right cellular context. In primary astrocytopathies such as Alexander disease, astrocyte-intrinsic mutations demonstrate that loss of a single astrocytic module can initiate pathology [17], while in complex disorders such as AD, PD, ALS, and HD, however, it remains less clear when astrocyte homeostatic functions are first lost, whether they are initial drivers or their dysfunction rather emerges alongside aggregate load, microglial activation, and neuronal injury [10]. This distinction matters therapeutically since early interventions may need to preserve homeostatic functions, whereas later approaches focus on interrupting self-sustaining inflammatory, excitotoxic, or proteostatic loops. Core translational hurdles, including heterogeneity and timing, targeting specificity, and the gap between model systems and human disease, remain the field’s most immediate practical constraints. Astrocyte states vary across regions and disease stages, meaning global suppression of reactivity risks dampening beneficial metabolic or antioxidant functions while failing to engage late toxic programs [26, 31, 38]. Moreover, astrocyte-selective delivery in humans remains imperfect despite advances in astrocyte-tropic AAV capsids and ALDH1L1/GFAP promoters, which still show off-target transduction and promoter leakage outside rodent systems [20, 166]. Finally, many mechanistic insights originate from mouse studies and human iPSC systems, candidate targets should be validated against human reference atlases such as the BRAIN Initiative Cell Census Network (BICCN) and the Human Cell Atlas (HCA). Without this cross-species anchoring, even well-supported targets risk being species or model-specific artefacts. Yet, these challenges also create a clear translational logic. Spatial omics identifies the niches where astrocytes converge on recurrent pathways of dysfunction, including EAAT2-mediated glutamate transport, Kir4.1/AQP4-dependent ion-water homeostasis, lysosomal-autophagic tone via TFEB or GBA1 and inflammatory modules centered on NF-κB, JAK/STAT3, and insufficient NRF2 counterprograms, and directly links these axes to vulnerable neuronal populations. These niche-specific signatures serve as mechanistic coordinates for therapy design. Importantly, spatially defined astrocyte states likely operate not as isolated cellular phenotypes, but as components of locally coupled astrocytic syncytia. Thus, spatial omics may identify where disease-associated states emerge, whereas connexin-dependent coupling may shape how ionic, metabolic, and homeostatic signals are coordinated or disrupted across local astrocyte territories [72, 177, 178]. Rather than targeting astrocytes globally, therapeutic strategies may therefore need to address both cell-intrinsic pathways and higher-order network functions within disease hotspots, such as (1) plaque-adjacent complement- and lysosome-high astrocytes in AD [10, 104, 107, 153], (2) CD44-high lysosomal-stressed astrocytes in the substantia nigra in PD [81, 125, 154], (3) EAAT2-low, Kir4.1-depleted ventral horn astrocytes in ALS [61, 93, 131], or (4) striatal zones in HD combining glutamate transport deficits with metallothionein-poor islands [11, 12, 68, 137]. Rare astrocytopathies further sharpen this logic: Alexander disease, VWM, and MLC demonstrate how disruption of individual astrocytic modules can generate spatially patterned degeneration and expose gaps in delivery and biomarker strategy [16, 96, 99, 143].

**Fig. 4:**
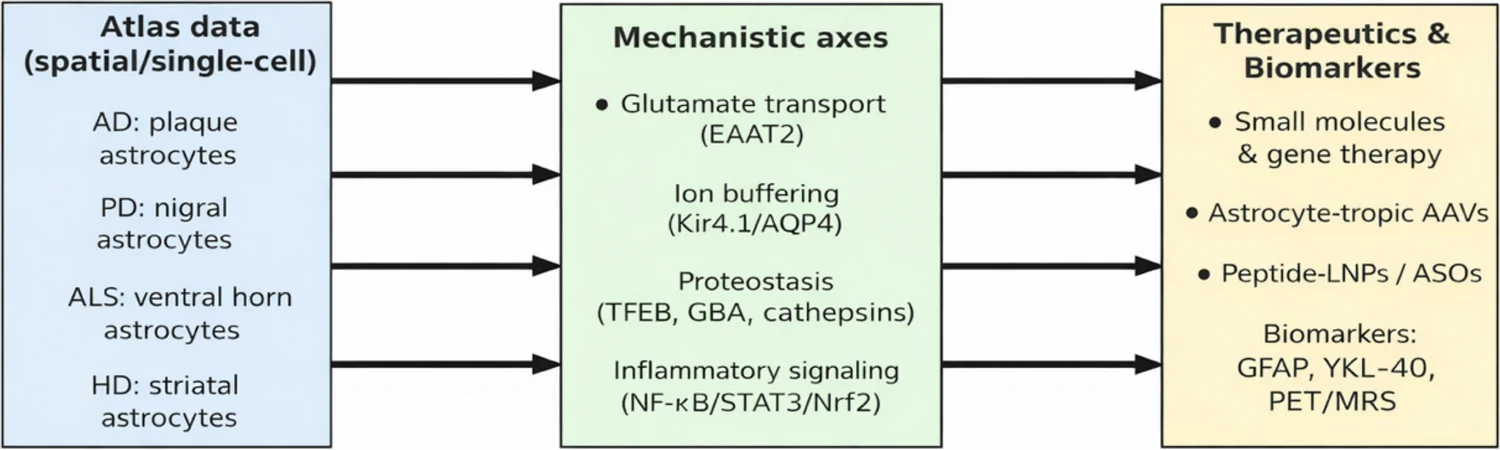
**From atlas data to therapeutic action: a translational plan for astrocytes.** Atlas-level datasets (left) define disease- and region-specific astrocyte states across Alzheimer’s disease (AD, plaque-adjacent astrocytes), Parkinson’s disease (PD, nigral astrocytes), amyotrophic lateral sclerosis (ALS, ventral horn astrocytes), and Huntington’s disease (HD, striatal astrocytes). These spatial and single-cell signatures converge on four mechanistic axes (middle) - glutamate transport (EAAT2), ion buffering (Kir4.1/AQP4), proteostasis (TFEB, GBA, cathepsins), and inflammatory signaling (NF-κB/STAT3/NRF2) - which represent recurrent points of vulnerability across disorders. Therapeutic strategies (right) can be mapped onto these axes: small molecules and gene therapies to restore glutamate transport or proteostasis, astrocyte-tropic AAVs and peptide-LNP/ASO platforms for selective delivery, and astrocyte-sensitive biomarkers (GFAP, YKL-40, PET/MRS) for patient selection and monitoring. Together, the scheme illustrates a practical “atlas-to-action” framework that links spatial omics, mechanistic anchors, and clinical translation

Accordingly, we here propose an “atlas → action” workflow for future astrocyte-targeted therapy development (see Fig. 4). First, human single-cell and spatial datasets define the pathogenic astrocyte state in the relevant anatomical territories. Second, these states are decomposed along the four mechanistic axes, guiding matched interventions like EAAT2 restoration, Kir4.1/AQP4 polarity repair, enhancement of lysosomal flux via TFEB or GBA, or selective dampening of NF-κB/STAT3-driven inflammatory programs [18, 122, 167, 169, 179, 180]. Third, delivery routes are aligned with spatial targets using astrocyte-biased promoters, regionally enriched AAV capsids, or peptide-functionalized LNPs, or intrathecal ASOs [17, 165]. However, this delivery step remains a major translational bottleneck. Although the mentioned platforms can enrich astrocyte engagement, none currently provides fully cell-type and region-restricted delivery in the human CNS, and delivery efficiency, biodistribution, and safety remain key constraints [20, 166, 181]. Finally, biomarker frameworks including plasma or CSF/plasma GFAP, YKL-40, MAO-B PET, or metabolic imaging provide in vivo confirmation of astrocyte engagement and enable patient stratification [109–111]. Taken together, spatial omics does more than refine our understanding of astrocytes, it provides a blueprint for intervention by prioritizing vulnerable niches rather than isolated astrocyte populations, identifying where astrocyte dysfunction occurs, which mechanisms dominate, and how targeted therapies can be deployed and monitored. In this sense, “atlas to action” provides a pipeline: a path from spatially resolved omics to precisely targeted therapies that act where astrocyte dysfunction is most tightly aligned with neuronal vulnerability.

## Conclusion

Astrocyte biology has undergone a fundamental transformation. Once viewed as passive responders to neuronal dysfunction, astrocytes are now understood as active, spatially patterned participants in neurodegeneration whose state transitions can precipitate, amplify, or constrain disease. Across neurodegenerative disorders and primary astrocytopathies, astrocytic failure consistently converges on a limited set of interacting mechanisms - disrupted glutamate handling, impaired ion-water homeostasis, lysosomal and autophagic stress, and maladaptive inflammatory signaling - that assemble into locally restricted pathogenic states. Spatial omics reframes therapeutic strategy by revealing that these states are not uniformly distributed but emerge within sharply defined anatomical niches shaped by local metabolic, proteostatic, and inflammatory pressures, providing a mechanistic framework for selective neuronal vulnerability. Rather than globally suppressing astrocyte reactivity, interventions can be designed to target specific state modules within vulnerable regions, enabling mechanistically rational and anatomically precise approaches. Rare monogenic astrocytopathies reinforce this logic by demonstrating that perturbation of single astrocytic modules is sufficient to generate regionally patterned degeneration, underscoring both the causal power of astrocytes and the need for spatially informed biomarker and delivery strategies. Moving from bench to bedside requires a translational workflow that begins with human single-cell and spatial datasets to define pathogenic states in the relevant anatomical territory. These states are then decomposed into their mechanistic axes, enabling targeted therapeutic strategies ranging from AAV-EAAT2 and Kir4.1 restoration to TFEB activation, GBA1 augmentation, or selective modulation of NF-κB/STAT3 inflammatory programs. Delivery modalities must reflect spatial targeting requirements, whether via astrocyte-biased promoters, regionally enriched capsids, or peptide-functionalized nanoparticles. Biomarkers such as CSF and plasma GFAP, YKL-40, MAO-B PET imaging, MRS-based glutamate measures, and emerging transcriptomic signatures anchor these interventions to verifiable astrocyte engagement.

In this integrated framework, spatial context of astrocytes becomes a guiding principle for clinical translation. Understanding exactly where astrocytes fail, which molecular pathways they engage, and how this aligns with neuronal vulnerability transforms astrocyte biology from descriptive pathology to actionable medicine. As mechanistic insight, spatial resolution, delivery platforms, and biomarker technologies converge, astrocyte-centered therapies are moving from conceptual promise to clinical reality, opening the possibility of modifying disease trajectories by restoring key homeostatic functions of astrocytes.

## Supplementary Information

Below is the link to the electronic supplementary material.

ESM 1 (DOCX 33.8 KB)

## Data Availability

All data supporting the findings of this study are available within the paper and its Supplementary Information. Data used for summarizing spatial analysis are found in Supplementary Table 1, along with all original references.

## Abbreviations

α-syn: α-synuclein
Aβ: Amyloid-β
AAV: Adeno-associated virus
AD: Alzheimer’s disease
ALDH1L1: Aldehyde dehydrogenase 1 family member L1
ALS: Amyotrophic lateral sclerosis
AMPA (fig.): α-Amino-3-hydroxy-5-methyl-4-isoxazolepropionic acid (receptor)
ApoE: Apolipoprotein E
AQP4: Aquaporin-4
ARE: Antioxidant response element
ASO: Antisense oligonucleotide
ATP13A2: ATPase cation transporting 13A2 (PARK9)
AxD: Alexander disease
BBB: Blood–brain barrier
BICCN: BRAIN Initiative Cell Census Network
C1q: Complement component 1q
C3: Complement component 3
C9orf72: Chromosome 9 open reading frame 72
CCL2 (fig.): C-C motif chemokine ligand 2
CD44: CD44 antigen (cell-surface glycoprotein)
cGAS: Cyclic GMP–AMP synthase
CLN: Ceroid lipofuscinosis, neuronal (gene family)
CNS: Central nervous system
CSF: Cerebrospinal fluid
Cx30: Connexin 30
Cx43: Connexin 43
CXCL10 (fig.): C-X-C motif chemokine ligand 10
DAMPs: Damage-associated molecular patterns
DJ-1: Protein deglycase DJ-1 (PARK7)
EAAT2/GLT-1: Excitatory amino acid transporter 2/glutamate transporter 1
eIF2B: Eukaryotic translation initiation factor 2B
FTD: Frontotemporal dementia
FUS (fig.): Fused in sarcoma
GABA: γ-Aminobutyric acid
GBA1: Glucosylceramidase beta 1 (β-glucocerebrosidase)
GFAP: Glial fibrillary acidic protein
Glc-6-P (fig.): Glucose-6-phosphate
GlialCAM: Glial cell adhesion molecule (protein encoded by HEPACAM)
GLUL: Glutamine synthetase
GSH (fig.): Glutathione
HCA: Human Cell Atlas
HD: Huntington’s disease
HEPACAM: Hepatic and glial cell adhesion molecule (gene encoding GlialCAM)
H_2_O_2_: Hydrogen peroxide
IL-1α/IL-1β: Interleukin-1 alpha/beta
iNOS (fig.): Inducible nitric oxide synthase
iPSC: Induced pluripotent stem cell
JAK/STAT: Janus kinase/signal transducer and activator of transcription
Kir4.1: Inwardly rectifying potassium channel 4.1
LNP: Lipid nanoparticle
LRRK2: Leucine-rich repeat kinase 2
MAO-B: Monoamine oxidase B
MAPK: Mitogen-activated protein kinase
MCT1/2/4 (fig.): Monocarboxylate transporter 1/2/4
mHTT (fig.): Mutant huntingtin
MLC: Megalencephalic leukoencephalopathy with subcortical cysts
MLC1: Megalencephalic leukoencephalopathy with subcortical cysts 1 (gene)
MMP (fig.): Matrix metalloproteinase
MRS: Magnetic resonance spectroscopy
NCL: Neuronal ceroid lipofuscinosis (Batten disease)
NF-κB: Nuclear factor kappa B
NLRP3: NOD-, LRR-, and pyrin domain-containing protein 3
NMDA-R (fig.): N-methyl-D-aspartate receptor
NRF2: Nuclear factor erythroid 2-related factor 2
PD: Parkinson’s disease
PET: Positron emission tomography
PHGDH (fig.): Phosphoglycerate dehydrogenase
PINK1 (fig.): PTEN-induced kinase 1
PPP (fig.): Pentose phosphate pathway
ROS: Reactive oxygen species
S100B (fig.): S100 calcium-binding protein B
SOD1: Superoxide dismutase 1
SPARC (fig.): Secreted protein acidic and rich in cysteine
STAT3: Signal transducer and activator of transcription 3
STING: Stimulator of interferon genes
TCA (fig.): Tricarboxylic acid (cycle)
TDP-43: TAR DNA-binding protein 43
TFEB: Transcription factor EB
TGF-β1: Transforming growth factor beta 1
TLR: Toll-like receptor
TNF: Tumor necrosis factor
TSPO: Translocator protein
UHDRS: Unified Huntington’s Disease Rating Scale
VEGF (fig.): Vascular endothelial growth factor
VWM: Vanishing white matter disease
YKL-40: Chitinase-3-like protein 1
