# Supplementary material for: Astrocytes in Neurodegeneration: Spatial States, Crosstalk, and Emerging Therapies
Source: Mol Neurobiol. 2026 Sep 14;63(1):893. doi: 10.1007/s12035-026-06142-x (PMC13574736; doi:10.1007/s12035-026-06142-x)
Supplement: Supplementary file 1 [file 12035_2026_6142_MOESM1_ESM.docx]

**Supplementary Data**

**Supplementary Methods and Table 1**

Supplementary Table 1 documents the reference basis for the qualitative evidence grading in Figure 2A. For each disease and mechanistic axis (glutamate homeostasis, ion/water buffering, proteostatic/lysosomal stress, and inflammatory-stress signaling), studies were selected for consistency, disease relevance, and mechanistic support of astrocytic involvement. The "+", "++", and "+++" scores reflect a consensus-based synthesis of converging neuropathological, experimental, transcriptomic, and imaging evidence — not a quantitative meta-analysis — with higher scores where astrocytic dysfunction is repeatedly observed, mechanistically linked to disease-relevant neuronal vulnerability, and supported across multiple lines of investigation.

In the "Main evidence base" column, "mixed" indicates support from more than one study type or model system (e.g. human postmortem, single-nucleus/spatial transcriptomics, patient-derived iPSC/organoid models, rodent functional or pharmacological studies, in vivo imaging) rather than a single approach. Qualifiers denote strength and directness: "strong" = convergent, reproducible, astrocyte-specific findings across independent studies; "indirect" = astrocytic involvement inferred rather than directly measured; "mainly model-/review-based" = support predominantly from animal/cellular models or review literature rather than primary human data.

Supplementary Table 1, References for Figure 2A

| Disease / axis | Relative prominence | Supporting literature | Main evidence base |
| --- | --- | --- | --- |
| AD – Glutamate homeostasis | ++ | Serrano-Pozo et al. 2024; Kuchibhotla et al. 2009; Wood et al. 2022 | Mixed, but partly indirect: human postmortem/snRNA-seq evidence for plaque-associated astrocyte states, AD mouse astrocyte calcium imaging, and systematic-review-level support for EAAT2/glutamate-transporter involvement in AD. |
| AD – Ion/water buffering | ++ | Iliff et al. 2012; Zeppenfeld et al. 2017 | Mixed: mouse glymphatic physiology plus human postmortem evidence linking perivascular AQP4 localization with cognition and Alzheimer disease. |
| AD – Proteostasis/lysosomal stress | +++ | Serrano-Pozo et al. 2024; Martini-Stoica et al. 2018; Nixon 2013; Xiao et al. 2014 | Mixed: human postmortem astrocyte-state data, astroglial tau uptake/TFEB evidence in mice, broader autophagy/lysosomal synthesis, and experimental support for astrocytic lysosome biogenesis in Aβ clearance. |
| AD – Inflammatory-stress signaling | +++ | Liddelow et al. 2017; Clarke et al. 2018; Serrano-Pozo et al. 2024; Mallach et al. 2024 | Mixed: model-based inflammatory astrocyte programs plus human/postmortem and spatial-omics support for reactive astrocyte states in AD. |
| PD – Glutamate homeostasis | + | Iovino et al. 2022; Das et al. 2025 | Mixed: human/mouse LRRK2-related evidence for impaired glutamate-transporter trafficking plus rodent pharmacological EAAT2 activation evidence. |
| PD – Ion/water buffering | + | Jia et al. 2025; Lopes et al. 2025 | Mainly review/model-based: glymphatic/AQP4 review-level evidence plus α-synuclein PFF mouse-model support for AQP4-linked α-synuclein propagation. |
| PD – Proteostasis/lysosomal stress | +++ | Aflaki et al. 2020; Loria et al. 2017; di Domenico et al. 2019; Rostami et al. 2017; Morrone Parfitt et al. 2024 | Mixed and strong: human iPSC-derived astrocytes, human astrocyte cultures, organoids, Gaucher/GBA-related astrocyte models, and in vitro α-synuclein uptake/transfer/degradation models. |
| PD – Inflammatory-stress signaling | ++ | Booth et al. 2017; Ma et al. 2025; Smajić et al. 2022; Liddelow et al. 2017 | Mixed: PD review-level evidence, human postmortem snRNA-seq/spatial-omics support, and broader model-based inflammatory astrocyte evidence. |
| ALS – Glutamate homeostasis | +++ | Rothstein et al. 1996; Bristol and Rothstein 1996 | Mixed and strong: experimental glutamate-transporter models plus human ALS postmortem motor-cortex evidence for altered glutamate-transporter expression. |
| ALS – Ion/water buffering | ++ | Kelley et al. 2018; Almad et al. 2022 | Mixed, but mainly ion/gap-junction based rather than water-specific: mouse Kir4.1 functional evidence plus ALS patient-derived and hiPSC astrocyte evidence for Cx43 hemichannel-mediated astrocyte toxicity. |
| ALS – Proteostasis/lysosomal stress | + / ++ | Maniatis et al. 2019; Clement et al. 2003 | Indirect mixed evidence: ALS mouse spatial transcriptomics, human postmortem ALS tissue, and SOD1 chimeric mouse evidence for non-cell-autonomous astrocyte involvement, but less direct lysosomal/proteostasis-specific astrocyte evidence. |
| ALS – Inflammatory-stress signaling | +++ | Endo et al. 2015; Maniatis et al. 2019; Sofroniew 2020 | Mainly mouse mechanistic evidence with human postmortem/spatial transcriptomic support and review-level synthesis; astrocyte-derived TGF-β1 and reactive astrocyte programs are especially well supported. |
| HD – Glutamate homeostasis | +++ | Diaz-Castro et al. 2019; Estrada-Sánchez and Rebec 2012; Jiang et al. 2016; Paryani et al. 2024 | Mixed and strong: HD mouse functional studies, corticostriatal/GLT1 dysfunction evidence, striatal astrocyte calcium and glutamate signaling defects, and human/postmortem omics evidence. |
| HD – Ion/water buffering | +++ | Tong et al. 2014; Jiang et al. 2016 | Mainly mouse functional/AAV evidence: strong support for astrocytic Kir4.1-related ion-buffering deficits, supported by broader HD astrocyte calcium/glutamate signaling and omics evidence; direct water-buffering evidence is less central. |
| HD – Proteostasis/lysosomal stress | ++ | Martinez-Vicente et al. 2010; Diaz-Castro et al. 2019; Paryani et al. 2024 | Mixed: cellular and mouse HD models, HD patient-derived/autophagy evidence, and human postmortem astrocyte/multi-omics evidence; partly disease-wide rather than exclusively astrocyte-specific. |
| HD – Inflammatory-stress signaling | ++ | Ben Haim et al. 2015; Al-Dalahmah et al. 2020; Bøstrand et al. 2024; Paryani et al. 2024 | Mixed: animal-model JAK/STAT3 astrocyte-reactivity evidence plus human postmortem snRNA-seq and multi-omics support for altered HD astrocyte states. |

**Supplementary Methods and Table 2**

Regional classifications in figure 3 were derived from peer-reviewed neuropathological, imaging, and transcriptomic studies referenced in Supplementary Table 2. Brain regions were categorized as “severe,” “impaired,” “mixed,” or “preserved” based on the consistency and strength of evidence for astrocytic dysfunction or regional pathology reported across studies.

“Severe” indicates robust and reproducible evidence of astrocytic dysfunction or neuronal vulnerability in the respective region; “impaired” reflects moderate but consistent alterations; “mixed” indicates heterogeneous or context-dependent findings; and “preserved” reflects absence of consistent regional involvement in the cited literature. Classifications were qualitative and consensus-based among the authors and were not derived from quantitative meta-analysis.

Supplementary Table 2, References for Figure 3

| Disease | Hippocampus / Entorhinal | Striatum | Substantia nigra | Spinal cord ventral horn | White matter | Cortex (general) |
| --- | --- | --- | --- | --- | --- | --- |
| **Alzheimer’s disease (AD)** | Braak & Braak 1991; Mathys et al. 2019; Serrano-Pozo et al. 2024 | Thal et al. 2002 | - | - | Sexton et al. 2011 | Zeppenfeld et al. 2017; Lau et al. 2023 |
| **Parkinson’s disease (PD)** | Halliday et al. 2014; Weintraub et al. 2015 | Braak et al. 2003; Patriat et al. 2022 | Fearnley & Lees 1991; Ma et al. 2025; Morrone Parfitt et al. 2024 | - | Patriat et al. 2022 | Halliday et al. 2014 |
| **ALS** | Bocchetta et al. 2018 | - | - | Kelley et al. 2018; Almad et al. 2022 | Agosta et al. 2010 | Tam et al. 2019; Li et al. 2023 |
| **Huntington’s disease (HD)** | Wilkes et al. 2023 | Tong et al. 2014; Jiang et al. 2016; Diaz-Castro et al. 2019; Paryani et al. 2024 | - | - | - | Rosas et al. 2008; Tabrizi et al. 2009 |
| **Alexander disease (AxD)** | - | - | - | - | Hagemann et al. 2006 | Hagemann et al. 2006 |
| **Vanishing white matter (VWM)** | - | - | - | - | Bugiani et al. 2010; Bugiani et al. 2018 | - |
| **Megalencephalic leukoencephalopathy (MLC)** | - | - | - | - | van der Knaap et al. 2012 | Bugiani et al. 2018 |
| **Batten disease (NCL)** | Nelvagal et al. 2020 | Nelvagal et al. 2020 | Nelvagal et al. 2020 | Nelvagal et al. 2020 | Nelvagal et al. 2020 | Nelvagal et al. 2020 |
